# Case of Anomalous Disease; with Appearances on Dissection

**Published:** 1816-09

**Authors:** 


### Medico-Chirurgical

### JOURNAL AND REVIEW.

VOL. II.] SEPTEMBER, 1816. [NO. 9.

### PART I.

### ORIGINAL COMMUNICATIONS.

——— quid utile ———

### *Case of Anomalous Disease; with Appearances on Dissection.*

By Mr. BAILEY.

GEORGE WHYATT, an athletic man of sanguine temperament, aged 50, had for several years been afflicted in such a way, as to make the history and description of his case difficult. It was one of those ailments that practitioners sometimes meet with; striking enough to shew, that the patient laboured under much suffering, but wearing no distinctive character so as to be nosologically defined. He had an anxious countenance; a dull sunk eye; thick white tongue; deficient salivary secretion; quick, and full pulse. Frequent cough; straightened respiration; rather tumid abdomen; regular bowels; no thirst; tolerable appetite. Pain of an obscure and indeterminate kind; sometimes in the legs, sometimes on the shoulders, but oftener in the right side; sickness at the stomach, with a wretched sensation of inward loss and sinking; rumbling, uneasy bowels, and frequent eructations of fœtid gas; wearisome days and toilsome nights; every chair and table in his cottage, and every gate and style in its neighbourhood, had occasionally served him to recline upon. Death at last assuaged his sorrows. It will be readily imagined, that to relieve thus much distress, various means were resorted to. Many practitioners in the neighbourhood were consulted, and each in his turn had a fair opportunity of applying his own remedies. It was curious and diverting enough to hear the reports of the patient upon the pathology of his case, as represented to him by his different advisers; there were few who did not regard the liver as the seat and cause of his ailment. There had, at a late period of his life, been some slight degree of intumescence in the region of the liver, but the skin and urine never shewed any appearance of obstructed bile. That grand, and now universal panacea, mercury, had been frequently resorted to and steadily persevered in, both internally and by friction. Leeches, cupping, perpetual blisters and setons were not omitted. In the latter years of the patient's life, I had not much to do with him; but I had twice attended him under most severe attacks of pleurisy, and I never could divest myself of the notion that, latterly, he laboured under some affection of the heart. A sensible practitioner who attended him last, positively rejected that idea, and insisted on the cause being seated in the liver. Salivation kept up gently for a month was to have cured him, but unfortunately he died sooner than was expected. It was always the wish of the deceased, that his body might be inspected post mortem; I accordingly opened him on the day of his death. The following is a correct account of appearances.

The stomach was empty and sound; the bowels were sound likewise, and contained a small portion of liquid fæcal matter; the bladder and kidnies were healthy; the pancreas was not so large as usual; the liver was *perfectly sound* and unaltered, both in colour and size; as far as one could judge from its appearance, it never had undergone any disease; the gall-bladder contained about an ounce of healthy looking bile; the spleen was gone, and seemed not to have performed its office for a long time; the mass substituted for this organ was about the size of a hen's egg, and resembled one somewhat in shape, and most exactly in colour; it was indurated to the texture of cartilage: at the lower apex it had approximated to bone, being with difficulty divided by a strong scalpel; it had no cavity, and shewed no vestiges of organization.

*Thoracic Cavity.*—When the cartilages of the sternum were divided, it was with extreme difficulty that it could be detached from the pleura, to which it adhered with astonishing firmness; this also was the case with that membrane in both cavities of the chest; so that it was impossible to get the lungs from their situation. The substance of these organs was, in like manner, considerably altered, having in many parts lost their cellular character, and become exceedingly dense and compact.

The heart was of its natural size, but adhered on its upper surface to the pericardium, which contained only a very small portion of fluid.

The obstinacy and indecisiveness of character of this long protracted case, and its similarity of progress and form to that of Stannard, a labouring man in the same parish, who died about the same time, urged me to recommend his relatives to allow Whyatt's body to be examined.—Stannard had gone, during life, nearly round the country in search of relief, but with as little success as his fellow sufferer. He was about 55 years old, but of more delicate form than Whyatt; had been jaundiced a time or two, and carried in his countenance the appearance of a person who had resided for some time in a tropical climate. In the early periods of his disorder, I had done something for him; but on his going to reside in the neighbourhood of another practitioner, I lost sight of him the last two or three years. His body was examined by his medical attendant at his own particular request, and from his daughter's report, there was nothing the matter with him, but water in the chest. I have never had an opportunity of ascertaining the nature of appearances from the person who made the examination.

*Harwich, July* 14, 1816.

